# Supplementary material for: Continuous emergence of phototaxis in Dictyostelium discoideum
Source: PLoS One. 2025 May 19;20(5):e0321614. doi: 10.1371/journal.pone.0321614 (PMC12088058; doi:10.1371/journal.pone.0321614)
Supplement: Table 1 — Cell densities are presented as the number of cells per square centimeter (cm2). Light and dark conditions were tested across multiple replicates and sub-replicates (PDF) [file pone.0321614.s001.pdf]

**Condition**	**Replicate**	**Sub-replicate**	**Number of cells/cm^2**
Light	1	1	1.6×10^7
		2	4×10^6
	2	1	6×10^6
		2	1.5×10^6
	3	1	1.6×10^7
		2	4×10^6
Dark	1	1	5×10^6
		2	2.6×10^6
	2	1	1×10^7
		2	2.6×10^6
	3	1	7.2×10^6
		2	2.4×10^6
	4	1	1.65×10^7
		2	8.25×10^6
