# Supplementary figures and images for: Continuous emergence of phototaxis in Dictyostelium discoideum
Source: PLoS One. 2025 May 19;20(5):e0321614. doi: 10.1371/journal.pone.0321614 (PMC12088058; doi:10.1371/journal.pone.0321614)

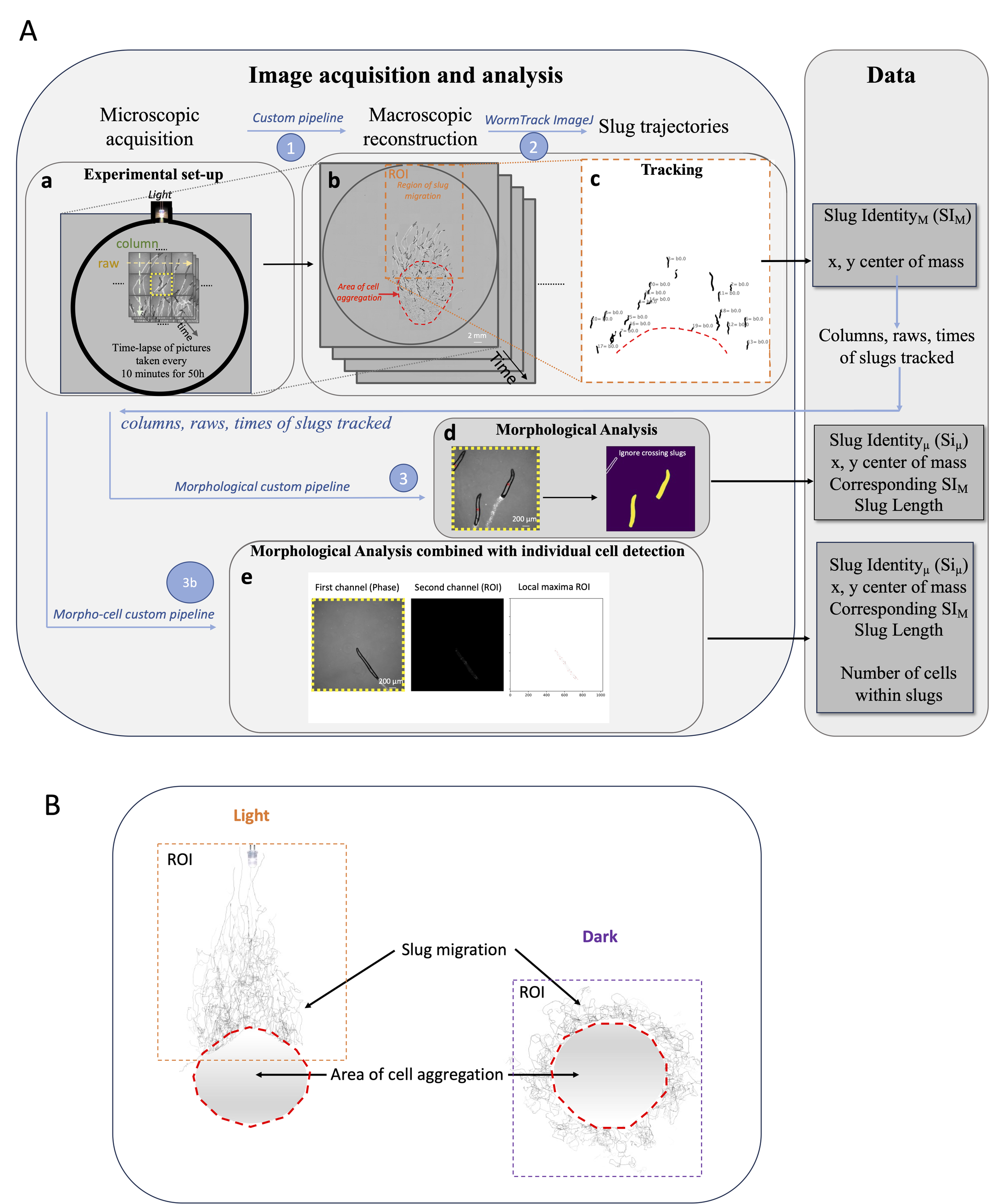

Supplement: S1 Fig — (A) Macroscale and microscale image analysis. a: Time-lapse acquisition was performed every 10 minutes for 50 hours using a 5X objective and phase contrast. b: Macroscopic reconstruction (1) from images taken at the microscale. The orange dashed rectangle corresponded to the Region Of Interest where slugs migrated. The red dashed polygon represented the area of cell aggregation removed from the analysis. (c) Slugs were tracked with wrMTrck imageJ plugin (2) yielding slug trajectories, (x,y) coordinates. From these coordinates, we obtained corresponding columns, raws, and times to analyze microscale images and extract morphological data. d: Morphological analysis of images (3) at the microscale (dash yellow square in a). Slugs that crossed the border were not analysed. The corresponding slug ID at the microscale (IDμ) was assigned to the slug ID tracked at the macroscale (IDM). Image acquisition at the microscale (a), macroscopic reconstruction (b) combined with analysis (1,2,3) were used to obtained phototaxis data. e: Morphological and cell number within slugs analysis (3b) performed at the microscale. This experiment was performed using phase contrast and fluorescent images in order to determine the cell number within living slugs. Phase contrast channel (left), fluorescent channel (center), and corresponding local maxima (right) detected using local maxima detection from the skiimage Python library. ( B) Trajectories obtained from slug tracking with the wrmtrack imageJ plugin in light (left) or dark (right) conditions within Regions of Interest, after removing the area of cell aggregation (red hashed polygons). All experiments were analysed by selecting Region Of Interest including slugs trajectories (orange and violet dashed rectangles for light and dark condition respectively). (TIFF) [file pone.0321614.s002.tif]

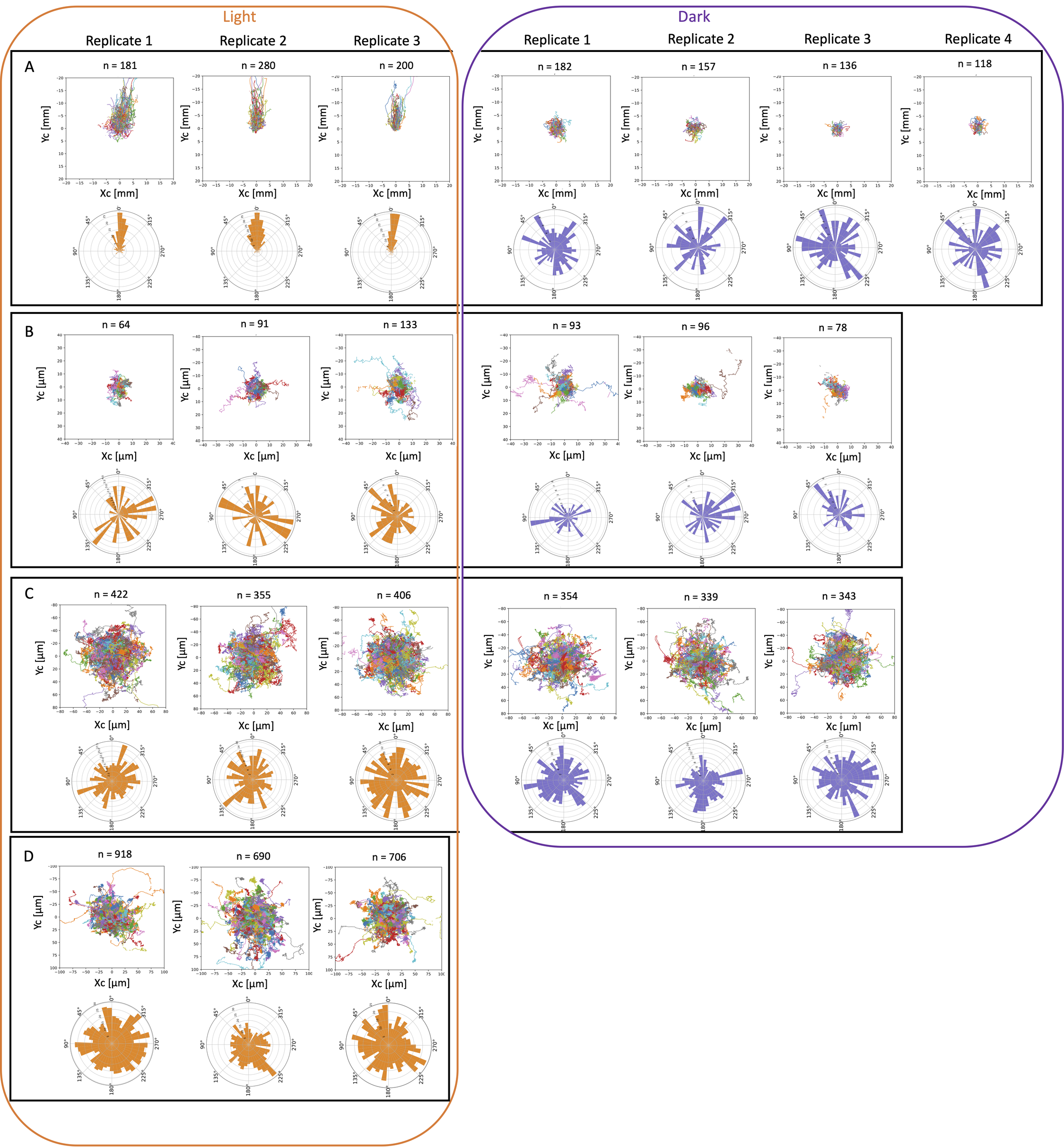

Supplement: S2 Fig — Slugs moved towards the light source (A). Cells from dis-aggregated slugs (B), vegetative cells at low density (C), and cells plated at high density (D) and exposed to light did not display any phototactic behaviour. (TIFF) [file pone.0321614.s003.tif]

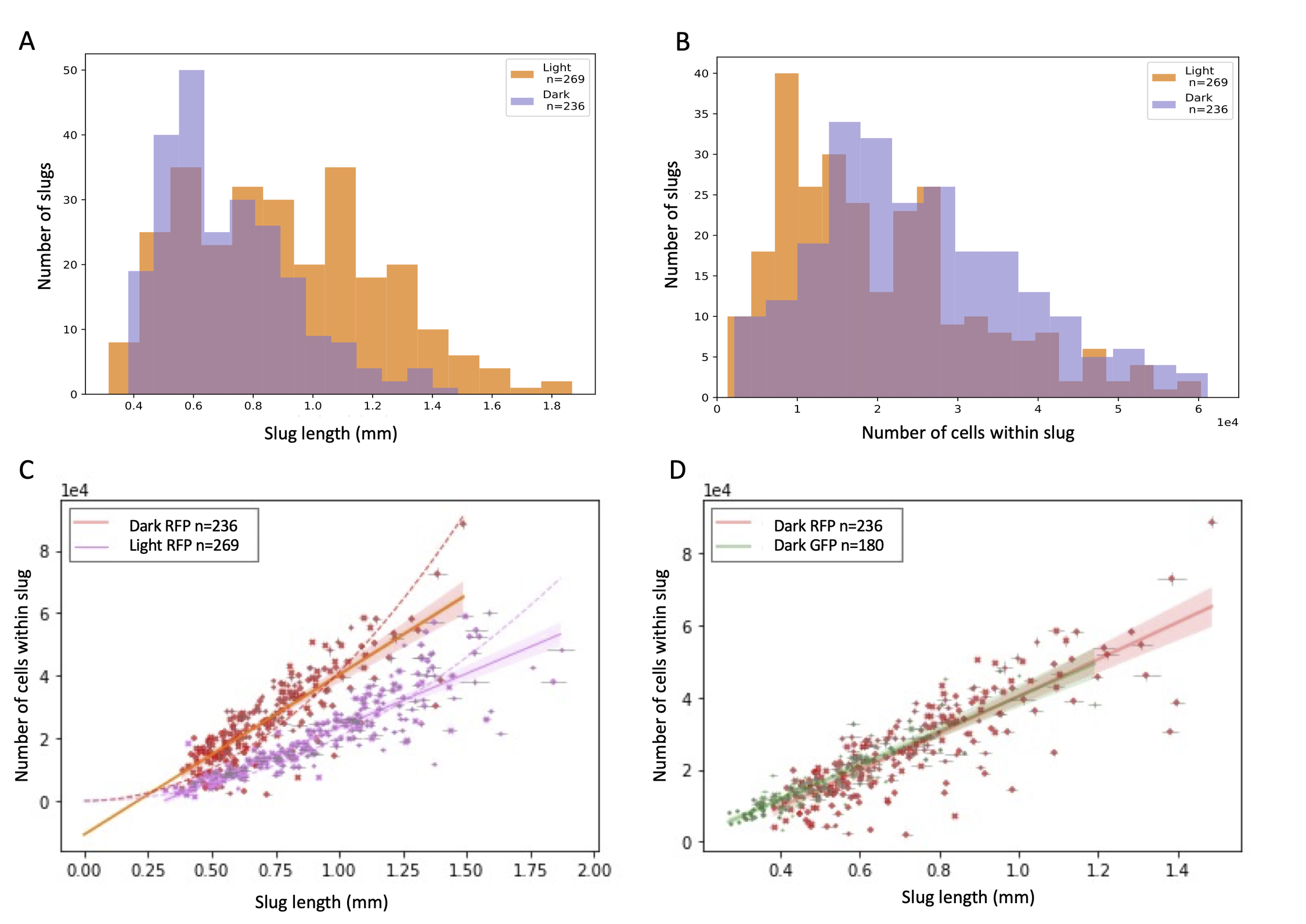

Supplement: S3 Fig — (A) Distribution of slug lengths when exposed to lateral light or in the dark. Slugs migrating towards lateral light exhibit a wider length range compared to slugs migrating in the dark. When slugs were exposed to lateral light, we observed significantly higher mean slug length compared to slug migration in the dark (0.90 mm ±0.32 mm and 0.71 mm ±0.22 mm in light and dark conditions respectively, Wilcoxon test p-value<10−4). Light induced slug elongation such that slugs with the same number of cells (B) were longer when exposed to light (ANOVA length * number of cells p-value=0.0182). (C) Slug length correlation when slugs were exposed to lateral light (magenta): cell number = 34 252 * length - 10 681 and in the dark (red): cell number = 51 085 * length - 10 749. For small length values, the correlation was fitted using a polynomial equation (dashed lines), for light: cell number = 20 409 (length)2 ; for dark: cell number = 41 194 (length)2. (D) Slug length correlates with the number of cells whatever the fluorescent marker (GFP in green or RFP in red) Each point corresponds to one slug, lines correspond to linear regression, dashed lines to polynomial fit. (TIFF) [file pone.0321614.s004.tif]

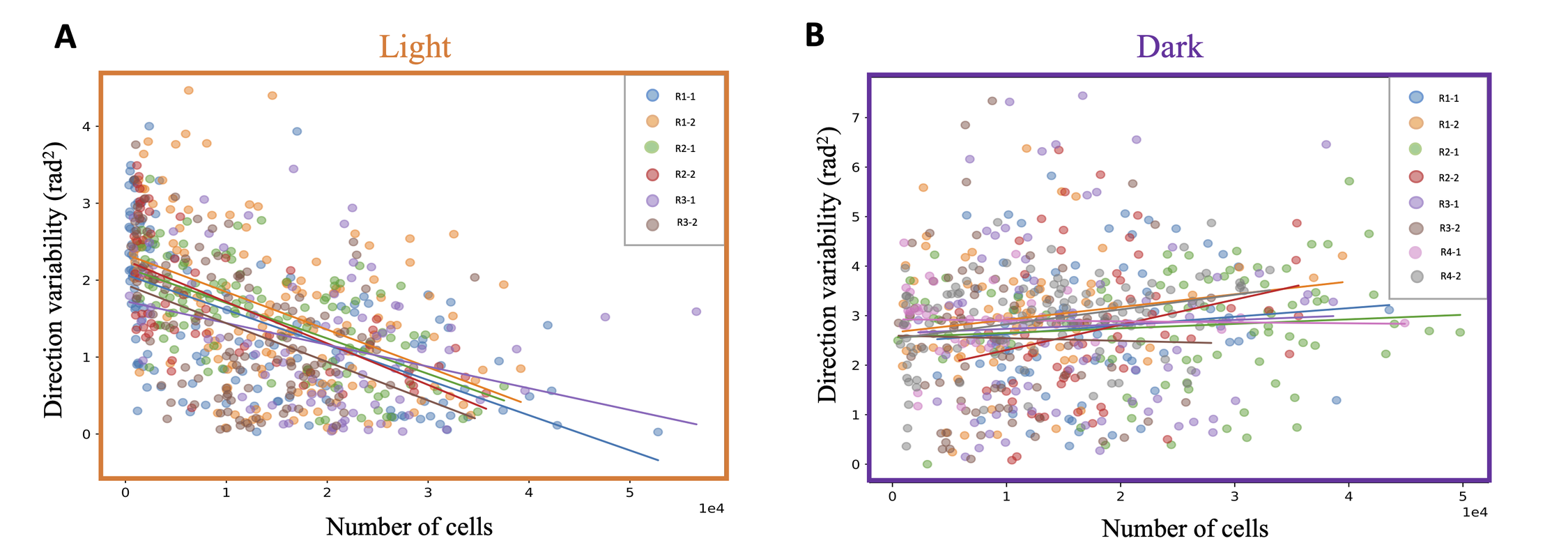

Supplement: S4 Fig — When slugs migrated towards light (A) direction variability decreased significantly with the increased number of cells (p-value<10−3) within slugs for all replicates. When in the dark (B), direction variability displayed no variation (p-value>0.05) with the number of cells within slugs. No significant difference was observed between replicates and sub-replicates (ANOVA number of cells*replicate-sub-replicate, light: p-value=0.48; dark: p-value=0.55). (TIFF) [file pone.0321614.s005.tif]

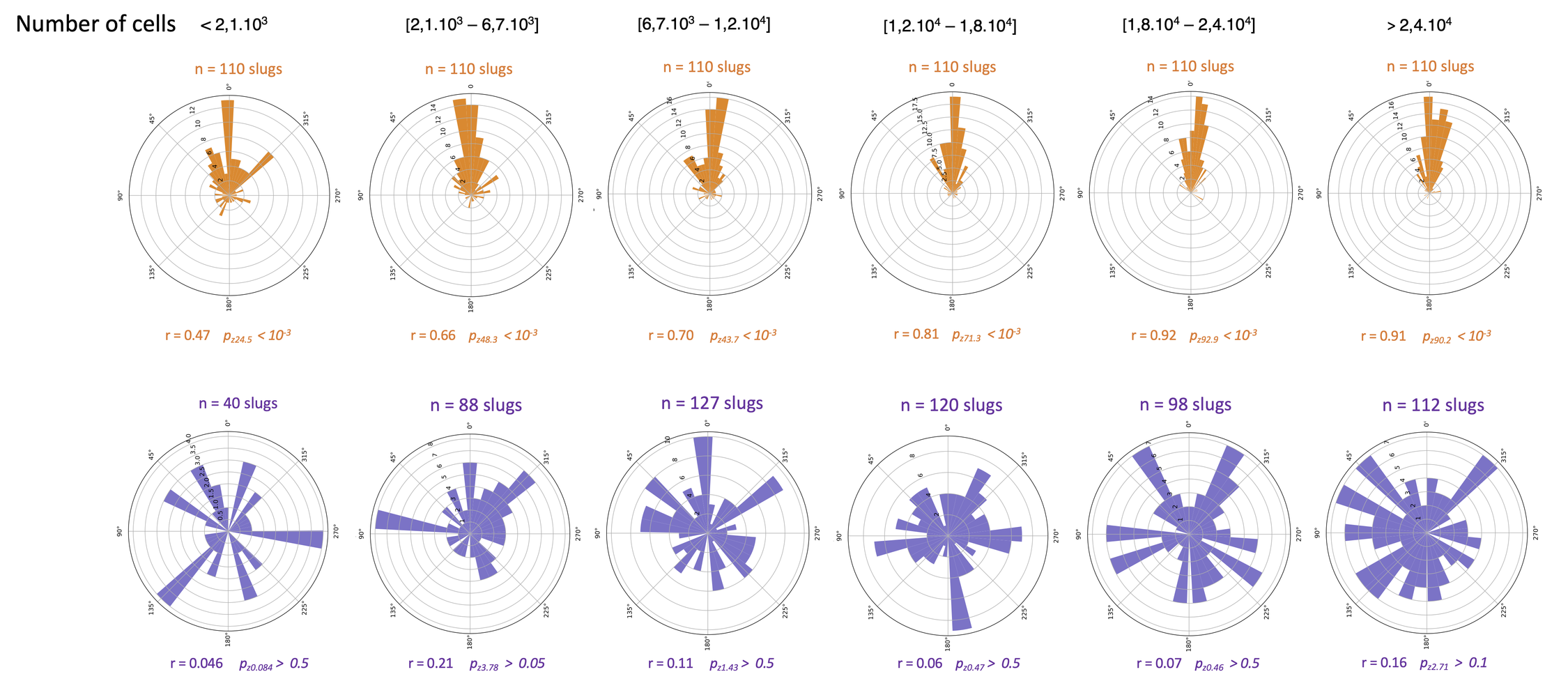

Supplement: S5 Fig — Each interval was composed of an equal and sufficient number of slugs for statistical analysis. Large slugs exhibit directional migration when exposed to light (upper line, orange), compared to dark conditions (violet). The r value computed from Eq (13) ranged from 0.046 to 0.21 and 0.47 to 0.91 for dark and light conditions respectively (from lower to higher slug sizes). (TIFF) [file pone.0321614.s006.tif]

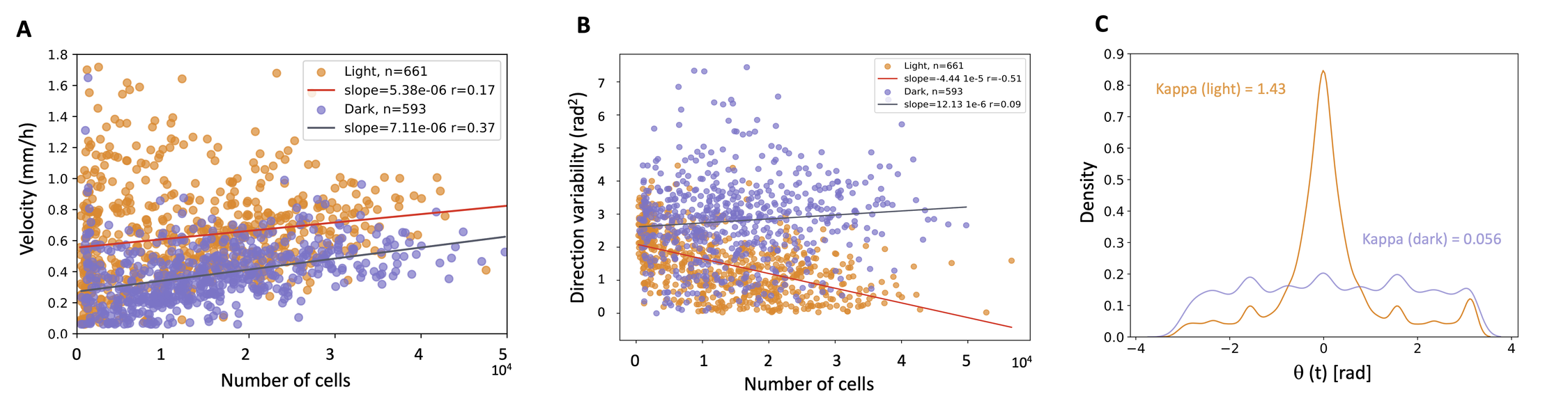

Supplement: S6 Fig — We observed no significant differences on slug speed between dark and light conditions (0.38 mm/h ±0.007 mm/h and 0.63 mm/h ±0.01 mm/h for dark and light conditions respectively, ANOVA number of cells: p-value<10−4, light: p-value<10−4, number of cells * light: p-value=0.8). Therefore, the slug speed was independent of the presence of the light but dependent of the number of cells within slugs. Previous works studying the light effect on slug speed have yielded conflicting results. Some authors reported an increase in slug speed following light irradiation [12,41,42] whereas others reported no changes on slug speed upon light [13,43]. ( A) Slug velocity for slugs exposed to light or in the dark. Slug velocity increased with the number of cells whatever the illumination condition (light: orange or dark: violet) and exhibited same slope whatever the illumination condition. ( B) Instantaneous direction variability (calculated following Eq (4)) as a function of cell number within slugs when in the dark (violet) and exposed to light (orange). Larger slugs exhibited a more linear trajectory when exposed to lateral light, but not in the dark. ( C) Distribution of instantaneous directions for slugs exposed to light (orange) or in the dark (violet). Corresponding kappa values quantifying bias of migration (following Eqs (2) and (5)). (TIFF) [file pone.0321614.s007.tif]

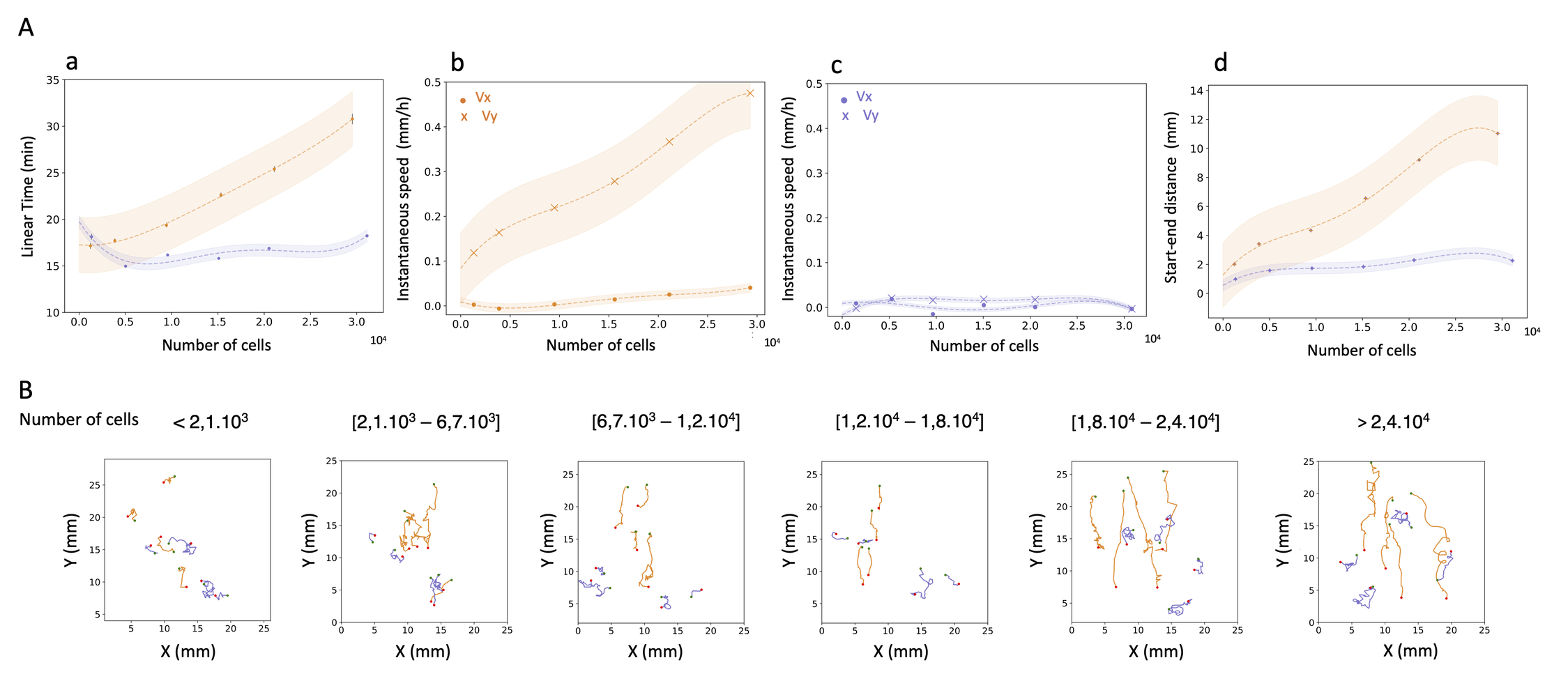

Supplement: S7 Fig — Linear time (A-a), parallel and orthogonal instantaneous velocity components (A-b for light and c for dark, from Eq (8)) and start-end distance (A-d computed from Eq (10)). All of these analyses showed continuously increasing phototaxis with slug size. In (B) examples of slug trajectories for each category of slug sizes. Slugs with small number of cells migrated at the same distance compared to slugs in the dark. Slugs migrating in the dark did not explore very far, whatever the number of cells inside slugs (violet) (for the same ranges as Fig 3). (TIFF) [file pone.0321614.s008.tif]
